# Analysis of somatic cell count, bacteria distribution and antimicrobial susceptibility in lactating dairy cows from small holder dairy farms in Kenya

**DOI:** 10.1016/j.onehlt.2026.101392

**Published:** 2026-03-28

**Authors:** Eugine L. Ibayi, Linnet Ochieng, Lydiah Kisoo, Nanna S. Hansen, Rikke L. Jensen, Sharon Anyona, Abdullahi M. Abdi, Arshnee Moodley, Dishon M. Muloi

**Affiliations:** a Health Program, International Livestock Research Institute, P.O. Box 30709, Nairobi 00100, Kenya; b Institute of Infection, Veterinary and Ecological Sciences, University of Liverpool, Leahurst Campus, Neston CH64 7TE, United Kingdom; c Department of Veterinary and Animal Sciences, University of Copenhagen, Grønnegårdsvej 15, DK-1870 Frederiksberg C, Denmark

**Keywords:** Mastitis, Risk factors, Somatic cell count, Small-holder dairy farms, Antimicrobial resistance

## Abstract

Subclinical mastitis is a major cause of economic loss and antimicrobial use in dairy cattle globally. In Kenyan smallholder systems, limited evidence on somatic cell counts (SCC), mastitis pathogens, and antimicrobial resistance patterns constrains effective control strategies. We conducted a cross-sectional study of 320 lactating cows from 119 smallholder farms in a peri-urban Kenyan county. Quarter milk samples (*n* = 1261) were cultured for *Escherichia coli*, *Klebsiella* spp., *Staphylococcus aureus*, and *Streptococcus* spp. and confirmed by MALDI-TOF MS. SCC was measured in 863 samples. Antimicrobial susceptibility testing was performed, and regression models used to identify animal- and farm-level factors associated with SCC variation. Elevated SCCs were associated with reduced teat-end clearance (< 30 cm, β = 3.1, *p* = 0.04), mid-lactation (β = 1.56, *p* = 0.03) and late-lactation (β = 1.7, *p* = 0.02), and higher parity (≥ 4 lactations; β = 2.1, *p* = 0.005). At farm level, increased SCC was associated with wet or muddy floors (β = 2.16, *p* = 0.001) and routine antimicrobial dry-cow therapy (β = 1.59, *p* = 0.02). Of 1251 samples, 67.8% showed growth on blood agar and 1% on MacConkey agar. *Streptococcus agalactiae* predominated (72.1%), followed by *Streptococcus uberis* (10.0%), *Staphylococcus aureus* (2.1%), and *Escherichia coli* (4.2%). *Streptococcus* spp. were susceptible to penicillins (100%), while tetracycline resistance was widespread in *Streptococcus* spp. (89.8%), *Staphylococcus aureus* (100%), and *Escherichia coli* (33.3%). Integrated control strategies targeting milking hygiene, environmental management, and antimicrobial stewardship are essential to reduce infection pressure and sustain productivity.

## Highlights

• Udder health management was suboptimal on Kenyan smallholder dairy farms.
• Somatic cell levels were influenced by host and farm-level factors.
• Contagious and environmental pathogens co-dominated infections.
• Most isolates remained susceptible to first-line antimicrobials.

## 1 Introduction

Mastitis poses a significant economic burden to global dairy production, leading to reduced milk yield, poor milk quality, and accounting for over 80% of antimicrobial use in dairy cows (1,2). While it is well known that mastitis can be substantially reduced – and numerous prevention and control programs have been implemented – it remains a major challenge in dairy production, compromising animal welfare, increasing veterinary costs, and reducing farm profitability (3,4).

Kenya has one of the largest and fastest-growing dairy sectors in sub-Saharan Africa, valued at approximately USD 1.7 billion and contributing 6–8% of national GDP. The sector supports 2.6 million smallholder farmers and 1.2 million jobs, with about 5 million milking cows producing 5.5 billion litres annually (5,6). Smallholders, typically managing two to fifteen cows, account for more than 80% of the national cattle population while contributing over half of the country's total dairy output (6). However, mastitis control in these systems is constrained by limited biosecurity investment, reliance on curative rather than preventive approaches, weak access to diagnostics and treatment guidance, and low farmer motivation ([7], [8], [9]). As a result, existing studies in Kenya report a high prevalence of subclinical mastitis (SCM), defined as intramammary infections without visible changes in milk, udder and animal physiology, ranging from 44%–92% (7,[10], [11], [12], [13]). These estimates are comparable to findings from a recent systematic review of 258 studies across 11 African countries, which reported a pooled SCM prevalence of 48.2%, with East African countries exhibiting the highest regional prevalence at 67.7% (14). By contrast, clinical mastitis, marked by noticeable changes in milk, udder and cow physiology, shows a lower prevalence among Kenyan small holder farms, typically between 0.9% and 6.8% (7,10,15,16). This large disparity reflects substantial under-detection of infection at the farm-level and highlights the dominant role of subclinical disease in driving productivity losses and antimicrobial use. In response to reduced milk yields, farmers often resort to non-selective or blanket antimicrobial treatment, particularly through widespread use of intramammary tubes. When infections persist or recur, veterinary intervention, if sought, often involves parenteral antimicrobial administration without microbiological confirmation (3,17,18). Such practices exert strong selective pressure for antimicrobial resistance, a critical and growing threat to both animal and public health.

Effective control of mastitis requires robust epidemiological data on prevalence and risk factors (19). In Kenya, a growing body of research has examined cow- and herd-level determinants of SCM; however, most studies have relied on the California Mastitis Test (CMT), a qualitative diagnostic method (20). Although somatic cell count (SCC) is globally recognized as a key indicator of udder health, milk quality and intramammary infection dynamics, recent SCC-based data from Kenyan dairy systems are lacking. The reliance on qualitative diagnostics limits benchmarking of udder health status, comparison across production systems, and evaluation of intervention effectiveness.

Kenya's dairy regions span a diverse range of farming systems from zero-grazing units to open pasture, and climatic zones, including temperate highlands and semi-arid lowlands (6,21). These ecological and management gradients strongly influence mastitis epidemiology. Clinical mastitis cases are most associated with *Staphylococcus aureus*, *Streptococcus* spp., and coagulase-negative *Staphylococcus* (CNS) (10,22,23). In contrast, CNS, *Staphylococcus aureus* and *Micrococcus* spp. predominate in SCM (16). Knowledge of pathogen-specific distributions is essential for guiding targeted treatment and antimicrobial stewardship; however, antimicrobial susceptibility data for mastitis pathogens in Kenya remain limited. Recent studies reported resistance rates to beta-lactams and sulfonamides ranging from 50% to 80%, with notably lower resistance (< 10%) to gentamicin and fluoroquinolones (24,25). To date, few studies have integrated quantitative SCC measurements with pathogen identification and antimicrobial resistance profiling at the farm level ([26], [27], [28]), limiting insight into mastitis infection dynamics and evidence-based control in smallholder systems.

The objective of this study was to analyse SCC levels in dairy cattle from smallholder farms in Kenya, to identify animal- and farm-level factors associated with elevated SCC, to characterize the distribution of mastitis-associated bacteria, and to assess their antimicrobial susceptibility profiles. By integrating quantitative SCC measurements with microbiological and resistance analyses, this study provides a detailed assessment of mastitis burden, pathogen ecology, and resistance phenotypes. This integrated approach strengthens the evidence base for targeted mastitis control strategies and antimicrobial stewardship in smallholder dairy systems in Kenya and other comparable settings.

## 2 Materials and methods

### 2.1 Study site and population

A total of 119 farms from the central Kenyan highlands in Githunguri Sub-County, Kiambu County (latitude −1.0586336; longitude 36.7779108; Supplementary Fig. S4), were enrolled in this study between September to December 2023. Kiambu County is a peri-urban region near Nairobi and is one of the major dairy-producing areas in Kenya (29,30). Dairy farming in this area is dominated by smallholder commercial farmers who typically keep between 5 and 50 cows on plots averaging 1–5 acres. Most cows are exotic breeds, primarily Friesian, Ayrshire, Guernsey, and their crosses, with an average daily milk production of 15–20 l per cow (31). We targeted farmers affiliated with the largest dairy cooperative in Kiambu County, which is also the largest in the country. As most smallholder dairy farmers in Kenya belong to farmer-owned cooperatives that provide access to extension services, markets, and key agricultural inputs, our study population is broadly representative of those engaged in cooperative-based dairy systems.

### 2.2 Sample size calculation and farm recruitment

The sample size was anchored in a pilot evaluation study assessing the field performance of a Point of Cow mastitis test (PoCt, an on-farm rapid milk test for distinguishing between Gram-positive and Gram-negative bacteria) in smallholder dairy systems in Kenya. For that study, the required number of independent lactating cows was calculated using Buderer's formula (32) for sensitivity and specificity, assuming a mastitis prevalence of 48% (33), test sensitivity of 88%, specificity of 97%, 5% precision, and 90% confidence, yielding 238 animals. Because animals were sampled within farms, clustering was accounted for using an intra-cluster correlation coefficient of 0.25 (34) and an average of three animals per farm. This produced a design effect of 1.5, resulting in an adjusted requirement of 357 cows, equivalent to 119 farms. The PoCt evaluation could not be completed as planned, but the original sampling and testing framework and calculated sample size were retained for the present analyses. Target study farms were randomly selected from the cooperative registry using the Microsoft Excel random number generator. Selected farmers were contacted with the assistance of the dairy cooperative extension officers, and written informed consent was obtained prior to participation.

### 2.3 Data collection and california mastitis testing

Upon enrolment, information was collected from the farmer or farm manager at each participating farm, including farmer and farm socio-demographics, cattle shed design, biosecurity practices, milking practices, and animal health practices (Supplementary Material S1). Data were collected using a standardised and validated questionnaire coded in Open Data Kit (ODK) software (35). The interview took approximately 30 min at each farm.

At each farm, three apparently healthy lactating cows, defined as having no visible or farmer reported recent milk, udder, or systemic abnormalities indicative of clinical mastitis, and without a history of recent antimicrobial use, were selected. Where only three lactating cows were present, all were included; on farms with more than three, cows were randomly selected. In five farms and 27 farms, only one cow and two cows were sampled, respectively, either because the other cows were clinically unwell or too aggressive to handle safely. In total, milk samples were collected from 320 lactating cows across 119 farms, representing 1261 udder quarters. Nineteen quarters from 17 cows could not be sampled because they had already been dried off (Supplementary Fig. S1). For each sampled cow, animal-specific information such as breed, age, average daily milk production, parity, and body condition score was collected (Supplementary Material S2).

Milk was sampled aseptically by field teams comprising veterinarians and veterinary paraprofessionals. Briefly, the udder and teats were washed and dried with disposable paper towels. Wearing clean gloves, the teats were cleaned with teat wipes (36). The first three streams of foremilk from each quarter were discarded. Milk from each selected quarter was then expressed into sterile 15 mL polypropylene vials and stored at 4 °C in insulated cooler boxes. Two aliquots were prepared: one for SCC and the other for microbiological testing. SCC testing was done within 12 h, and samples for microbiology were transported to the laboratory at 4 °C and processed within 8 h of collection.

CMT was performed on the farm using a reconstituted CMT reagent, according to the manufacturer's instructions. About 3 mL of milk directly from each quarter was collected using a CMT paddle and mixed with an equal amount of the reagent. The paddle was gently swirled, and gel formation was observed within 10–15 s, indicating the presence and severity of mastitis. Based on the degree of gel formation, results were interpreted as negative (0), weakly positive (1), distinctly positive (2), or strongly positive (3). All results were recorded into the ODK platform, and feedback was provided to farmers for each cow sampled.

### 2.4 Somatic cells count testing

SCC analysis was performed using a DeLaval Cell Counter (DeLaval, Tumba, Sweden), purchased and calibrated by the supplier (Tetra Pak) in 2023, coinciding with the start of testing. Cost reasons precluded SCC analyses on all samples; SCC was therefore determined for 863 quarters from 223 animals across 84 farms (Supplementary Fig. S1).

### 2.5 Bacteriological analysis

A total of 1261 milk samples from individual quarters were collected, of which 1251 were included in the final bacteriological analysis; ten samples were excluded at laboratory reception due to sample spillage during transportation or suspected mislabelling. Milk samples were processed using standard bacteriological methods for culture and isolation of four target primary mastitis-associated bacteria: *Escherichia coli*, *Klebsiella* spp., *Streptococcus* spp. and *Staphylococcus aureus*. Ten microlitres of each milk sample was plated on 5% sheep blood agar (BA) for isolation of *Streptococcus* spp. and *Staphylococcus aureus,* and on MacConkey (MAC) for isolation of *Escherichia coli* and *Klebsiella* spp. Plates were then incubated aerobically at 37 ± 1 °C for 24 h. A quarter was considered culture-positive if ≥1 colony- forming unit of any target pathogen was detected. Samples yielding a single colony morphology were classified as pure cultures, whereas those with ≥2 distinct morphologies were classified as mixed cultures. Mixed growth samples were not excluded as contamination. Instead, they were recorded as mixed cultures and processed further if at least one target pathogen was present. For each culture positive sample (pure or mixed), one presumptive colony per target pathogen was selected based on characteristic colony: *Escherichia coli* (pink), *Klebsiella* spp. (pink mucoid), *Staphylococcus aureus (*cream beta-haemolytic*)*, or *Streptococcus* spp. (tiny greyish white), and sub-cultured on BA and archived at −80 °C. Presumptive isolates were identified using matrix-assisted laser desorption/ionization time-of-flight mass spectrometry (MALDI-TOF MS) (37). Bacterial isolates were classified as contagious (*Streptococcus agalactiae, Staphylococcus aureus*) or environmental (*Escherichia coli,* other *Streptococcus* spp., *Klebsiella oxytoca*) pathogens.

### 2.6 Antimicrobial susceptibility testing

Antimicrobial susceptibility testing using Kirby-Bauer disk diffusion was performed on 206 *Streptococcus* spp. isolates (18 isolates were excluded due to loss of viability during storage) and all *Staphylococcus aureus*, following the guidelines of the Clinical and Laboratory Standards Institute (CLSI) VET01S 7th edition (38)**.** Broth microdilution for *Escherichia coli* and *Klebsiella oxytoca* was performed following the European committee on Antimicrobial Susceptibility Testing (EUCAST) guidelines (39). Mueller-Hinton agar and broth (Oxoid, UK) were used. The reference strains used for quality control included *Escherichia coli* ATCC 25922 for Gram-negative bacteria and *Staphylococcus aureus* ATCC 29523 for *Staphylococcus aureus.* Antimicrobials were selected based on the antimicrobials commonly used in Kenyan dairy farms. For *Streptococcus* spp.*,* penicillin G (10 μg), ceftiofur (30 μg), pirlimycin (2 μg), penicillin-novobiocin (40 μg), clindamycin (2 μg), erythromycin (15 μg), sulfamethoxazole/trimethoprim (25 μg), and tetracycline (30 μg) were tested, while for *Staphylococcus aureus*, ceftiofur (30 μg), pirlimycin (2 μg), penicillin-novobiocin (40 μg), clindamycin (2 μg), erythromycin (15 μg), tetracycline (30 μg), gentamicin (10 μg), sulfamethoxazole/Trimethoprim (25 μg), and cefoxitin (30 μg) were tested. For *Escherichia coli* and *Klebsiella oxytoca*, the Sensititre EUVSEC (Thermo Fisher Scientific, USA) panel was used. For disk diffusion, results were interpreted as susceptible, intermediate, or resistant using CLSI breakpoints. For broth microdilution (BMD), minimum inhibitory concentrations (MICs) were categorised as wild type or non-wild type using EUCAST epidemiological cut-off values (ECOFFs).

### 2.7 Statistical analysis

All analyses were conducted in R software (version 4.5.0) (40). We initially compared CMT results with SCC readings from 863 quarters across 223 animals from 84 farms. SCM was defined as a CMT score of >1 or an SCC ≥ 250,000 cells/mL. There was a lack of concordance between the two methods: 8% of quarters (70/863 from 38 cows) had SCC > 250,000 cells/mL but were CMT-negative (Supplementary Fig. S2). Owing to this discordance, SCC was used as the primary outcome variable for all subsequent analyses.

We applied generalised linear mixed-effects models (GLMM) to explore animal- and farm-level factors associated with variation in SCC. Animal-level variables included parity, milk yield on the previous day, reported history of mastitis, body condition score, teat-to-floor distance, and lactation stage. Farm-level variables comprised floor type, housing system, post-milking teat disinfection, udder washing with water, use of a strip cup for mastitis detection, use of a shared cloth udder towel across cows, a farm hygiene score, the moisture status of the cattle house floor (wet/muddy versus dry), complete stripping during milking, and whether cows were kept standing after milking. Definitions and rationale for all explanatory variables are detailed in Supplementary Table S1.

Two separate models were constructed: model 1 (animal-level) and model 2 (farm-level). SCC values were natural-log transformed. Farm and cow ID were included as random effects. To identify factors influencing SCC variation, we separately ran the same GLMM analysis for each model above. We then used the dredge function in the R MuMIn package for each model separately, which generated a set of models representing all possible combinations of initial variables and ranked them according to the Akaike Information Criterion (AIC). The top-ranked model with the lowest AIC value (∆AIC < 2) was selected as the most plausible to explain a substantial proportion of variance in the data. *P*-values were obtained from the lmerTest package, and standard errors and means were subsequently back-transformed to the original scale. For all statistical analyses, significance was considered at *P* ≤ 0.05. We assessed adjusted R^2^ as measures of the proportion of variance explained by the model, using the r.squared GLMM function from the MuMIn package. Model diagnostics indicated normally distributed residuals with no evidence of heteroscedasticity. Multicollinearity among fixed effects was assessed using the performance package, and all variance inflation factors were below two, indicating no evidence of collinearity. Separately, we analysed factors associated with contagious and environmental mastitis using univariable regression models. Proxy management practices were analysed for each mastitis type, including shared cloth udder towel use and hand hygiene for contagious mastitis, and floor moisture status for environmental mastitis.

## 3 Results

### 3.1 Farm demographics and characteristics

A total of 119 dairy farms took part in the study. Most respondents were farm owners (76%), most of whom were male (64% of owners and 96.4% of workers). The average herd size was 13.3 dairy cattle, including an average of 7.1 (range 3–31) lactating cows per farm. Among the 320 cows sampled, 96.2% (*n* = 308) were Friesian-Holstein, 1.9% (*n* = 6), Ayrshire, 0.3% (*n* = 1), Guernsey and 1.6% (*n* = 5) crossbreeds. The animals averaged 4.6 years in age (median 4.8 years, IQR 3–5) and produced a mean of 17.9 l of milk per day (median 18 l, IQR 13–22) (Table 1).

**Table 1:** Demographics of the dairy cattle farms that participated in the study.

Demographics	Descriptive statistics
Daily activity	*N* = 119
Farm owner	91 (76%)
Farm worker/manager	28 (24%)
Farm owner (*n* = 91)	
Gender	
Male	58 (64%)
Female	33 (36%)
Age, mean in years (range)	50 (23, 83)
Education	
No formal education	6 (6.6%)
Primary	23 (25.3%)
Secondary	32 (35.1%)
Tertiary	30 (33%)
Experience in farming in years, mean (range)	17.4 (1, 59)
Received any form of training on dairy cattle production, disease prevention or control	
Yes	75 (82.4%)
No	16 (17.6%)
Farm manager/worker (*n* = 28)	
Gender	
Male	27 (96.4%)
Female	1 (3.6%)
Age in years; mean (range)	30.9 (20, 63)
Education	
No formal education	1 (3.6%)
Primary	12 (42.8%)
Secondary	11 (39.3%)
Tertiary	4 (14.3%)
Duration in dairy farm management in years; mean (range)	6.1 (1,20)
Received any form of training on dairy cattle production, disease prevention or control	
Yes	12 (42.9%)
No	16 (57.1%)
Farm characteristics	
Total number of cattle kept; mean (range)	13.3 (3, 49)
Number of lactating cows; mean (range)	7.1 (3,31)
Cows sampled characteristics (*n* = 320)	
Age of sampled cows in years; mean (range)	4.6 (1.2, 12)
Amount of milk produced per day (l); mean (range)	17.9 (3; 41)
Parity of the sampled cows; mean (range)	2.4 (1,12)

### 3.2 Farm biosecurity and udder health measures

Nearly all farms (113/119, 95.0%) used open-shed housing. Disinfectant footbaths were present at only 11 farms (9.2%), and just 40 (33.6%) imposed any formal restrictions on visitor entry. Dedicated coveralls and boots for workers, visitors or service providers were required on 16 farms (13.4%). Almost all farms (95%, *n* = 113) had concrete floors, while the remaining 5% had an earthen floor. Whilst most farms (94.1%, *n* = 112) reported cleaning the floor daily, the surface was still visibly muddy on 78.2% of farms. Workers on five farms (4.2%) reported working on multiple dairy holdings. Hand milking was the main method on almost all farms (115/119, 97.6%), with only four using milking machines. All farms reported cleaning teats and udders of cows before milking: most (106/119, 89.0%) used water, eight (6.7%) applied a pre-dip disinfectant and five (4.2%) used a commercial wet towelette. After cleaning, 107 farms (90.0%) dried teats/udders with a reusable cloth udder towel, nine (7.6%) did not dry at all and three (2.5%) used disposable paper towels. Of the 107 farms reporting using reusable cloth udder towels, 60% (*n* = 64) reused the towel on multiple cows. Half the farms (60/119, 50.4%) reported using a strip cup before milking to screen for mastitis. Among the 115 hand-milking farms, all workers reported washing their hands before milking, but only 79 (68.7%) washed between cows. Nearly all farms (118/119, 99.2%) reported stripping the udder completely, and most kept cows standing after milking (111/119, 93.3%). Post-dip teat disinfection was practised on only 21 farms (17.6%).

### 3.3 Analysis of factors influencing variation in SCC level

**Fig. 1:**
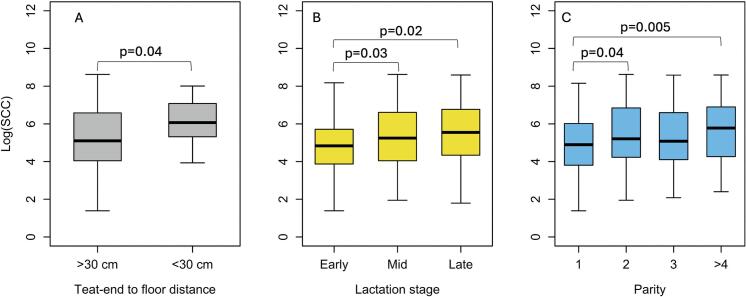
Boxplots showing association between somatic cell count (SCC) and animal level factors: A) teat end to floor clearance, B) lactation stage and C) parity.

**Fig. 2:**
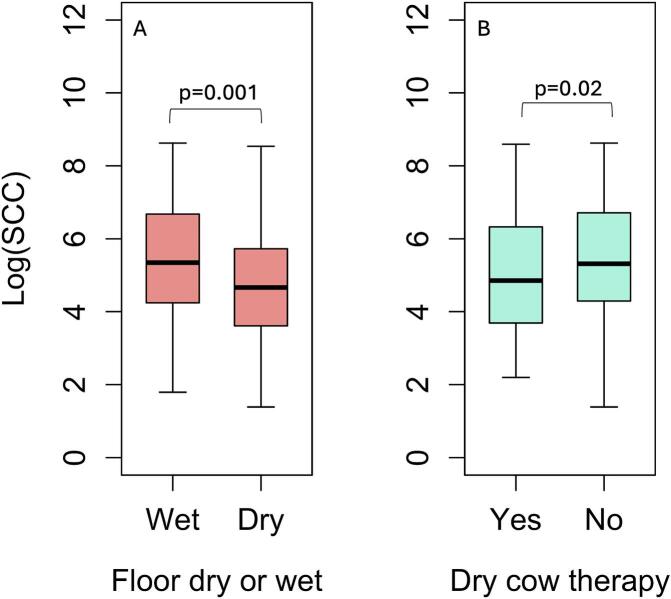
Boxplots showing association between somatic cell count (SCC) and farm level factors: A) floor condition and B) antibiotic use in dry cow therapy.

The median SCC was 169,000 cells/mL (IQR 58000–728,500). Higher SCC was associated with teat-end clearance <30 cm versus ≥30 cm (*p* = 0·04, β = 3.1, 95% CI = 1.05–9.32, Fig. 1A), with mid- (*p* = 0·03, β = 1.56, 95% CI = 1.04–2.35) or late- (*p* = 0·02, β = 1.7, 95% CI = 1.09–2.66) lactation versus early lactation (Fig. 1B), and with multiparity (Fig. 1C); especially second (p = 0·04, β = 1.54, 95% CI = 1.0–2.35) and ≥ 4 lactations (p = 0·005, β = 2.09, 95% CI = 1.25–3.5) compared with primiparous cows (Model 1, R^2^ = 55.2%, Table 2). At the farm level, SCC was significantly higher on farms with wet floors (p = 0·001, β = 2.16, 95% CI = 1.38–3.36 Fig. 2A) and likewise farms that routinely used antimicrobial dry-cow therapy (DCT) (p = 0·02, β = 1.59, 95% CI = 1.08–2.35, Fig. 2B) (Model 2, R^2^ = 29.3%, Table 2).

**Table 2:** Multivariable model coefficients for animal- and farm-level factors associated with somatic cell count.

Variable (reference level)	Level	Estimate (β)	95% CI	*P* Value
Model 1: Animal Level				
Parity (ref = 1)	2	1.54	1.0–2.35	0.04
	3	1.53	0.98–2.35	0.06
	≥4	2.09	1.253–3.5	0.005
Teat-end to floor distance (ref = ≥ 30 cm)	<30 cm	3.12	1.05–9.32	0.04
Lactation stage (ref = early)	Mid	1.56	1.04–2.35	0.03
	Late	1.7	1.09–2.66	0.02
Model 2: Farm Level				
Floor condition (ref = dry)	Wet	2.16	1.38–3.36	0.001
Antibiotic use in dry-cow therapy (ref = no)	Yes	1.59	1.08–2.35	0.02

Abbreviations: CI = Confidence Interval; ref. = reference; cm = centimetres.

### 3.4 Bacterial culture

Out of the 1251 milk samples cultured, 67.8% (848/1251) grew on BA, while 1% (13/1251) grew on MAC. Among the 848 BA-positive samples, 37.9% (322/848) yielded pure colonies presumptive of the target bacteria. In contrast, 1.3% (11/848) of BA-positive samples exhibited mixed growth, each with two morphologically distinct colonies, resulting in a total of 22 presumptive isolates from mixed cultures. Out of the 322 single presumptive isolates, 68.2% (220/322) were confirmed as target pathogens. Among the 22 presumptive isolates obtained from the 11 mixed-growth BA plates, 40.9% (9/22) were confirmed as target pathogens. Additionally, of the 13 milk samples that showed growth on MAC, 84.6% (11/13) were confirmed as target pathogens (Table 3, Supplementary Fig. S3). Of the target isolates, the most frequently isolated were *Streptococcus agalactiae* (72.1%, 173/240), for Gram-positive bacteria, and *Escherichia coli* (4.2%, 10/240), for Gram-negative bacteria. Notably, two milk samples grew both *Streptococcus* spp. and *Staphylococcus aureus*.

**Table 3:** Distribution of the target bacteria.

Bacterial pathogen	Blood agar with pure colonies	Blood agar with mixed colonies	MacConkey agar	Total
	(*n* = 220)	(*n* = 9)	(*n* = 11)	(*n* = 240)
**Contagious**				
*Streptococcus agalactiae*	167 (75.9%)	6 (66.7%)	–	173 (72.1%)
*Staphylococcus aureus*	3 (1.4%)	2 (22.2%)	–	5 (2.1%)
**Environmental**				
*Escherichia coli*	–	–	10 (90.9%)	10 (4.2%)
*Klebsiella oxytoca*	–	–	1 (9.1%)	1 (0.4%)
*Streptococcus dysgalactiae*	2 (0.9%)	–	–	2 (0.8%)
*Streptococcus gallolyticus*	11 (5.0%)	–	–	11 (4.6%)
*Streptococcus mitis/oralis*	4 (1.8%)	–	–	4 (1.7%)
*Streptococcus uberis*	24 (10.9%)	–	–	24 (10.0%)
*Streptococcus parasanguinis*	2 (0.9%)	–	–	2 (0.8%)
*Streptococcus parauberis*	5 (2.7%)	–	–	5 (2.1%)
*Streptococcus pseudoporcinus*	–	1 (11.1%)	–	1 (0.4%)
*Streptococcus salivarius*	2 (0.9%)	–	–	2 (0.8%)

### 3.5 Distribution of target pathogens among sampled cows and farms

**Fig. 3:**
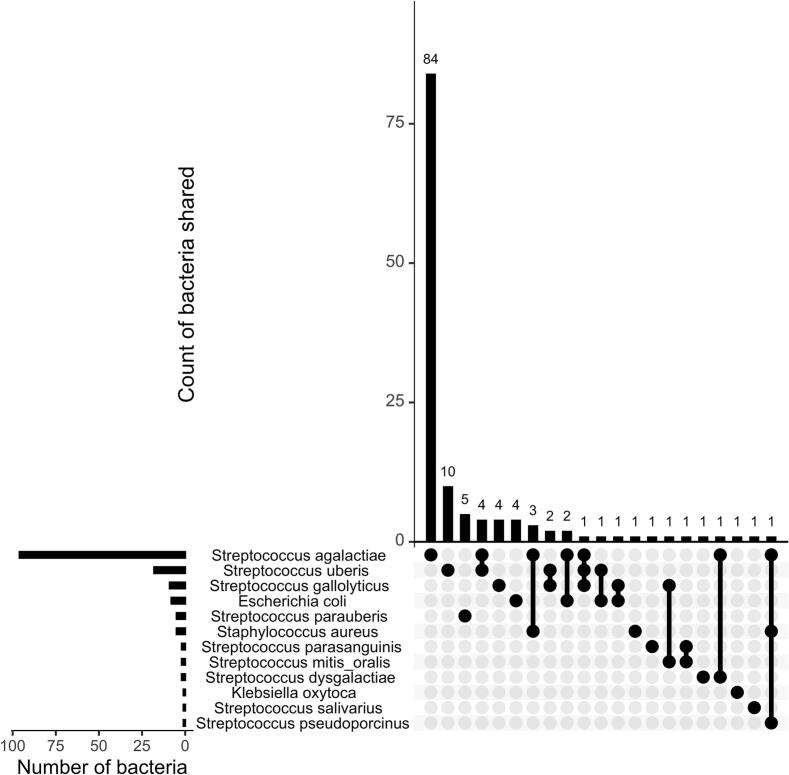
UpSet plot of distribution and co-occurrence of mastitis causing bacteria from milk samples in 320 cows.

Of 320 cows included in the study, the target pathogens were detected in 40.6% (*n* = 130) of the cows present in 83 farms (69.7%, 83/119). Most of the infected animals (86.2%, *n* = 112) carried a single pathogen, with *Streptococcus agalactiae* being the most common isolate, detected alone in 84 cows (75%). Other single-pathogen infections included *Streptococcus uberis* (*n* = 10 cows), *Streptococcus parauberis* (*n* = 5 cows), *Escherichia coli* (*n* = 4 cows), and *Klebsiella oxytoca* (*n* = 1 cow). Co-infections were observed in 13.8% (*n* = 18) of cows, most frequently *Streptococcus agalactiae* with *Streptococcus uberis* (*n* = 4) and *Streptococcus agalactiae* with *Staphylococcus aureus* (*n* = 3) (Fig. 3).

Similarly, at the farm level, most herds harboured a single pathogen (60/83), while multiple pathogens were detected in 23 herds. Of these, 16 farms harboured two pathogens, six farms harboured three pathogens, and one farm harboured four distinct pathogens. Next, we analysed the distribution of mastitis categories (contagious versus environmental) in relation to farm management practices. Overall, 68.5% (89/130) of animals with target pathogens from 65 farms were classified as having contagious mastitis only, 25.4% (33/130) from 33 farms had environmental mastitis only, and eight animals (6.1%) from eight farms presented with both contagious and environmental mastitis. No significant differences were observed between our proxy management factors influencing either mastitis type. An equal proportion of animals with contagious mastitis were found on farms where farmers reported sharing the same cloth udder towel (49.5%, 48/97) compared with those that did not. Similarly, nearly all animals with contagious mastitis were from farms (97%, 94/97) where farmers reported washing their hands before milking. In contrast, 78% (32/41) of animals with environmental mastitis were from farms with visibly muddy floors.

### 3.6 Antimicrobial susceptibility

Among *Streptococcus* spp. isolates, 8.7% (18/206) were fully susceptible to all antimicrobials tested. Resistance was highest to tetracycline (89.8%, 185/206), with lower resistance rates to erythromycin (9.2%) and clindamycin (5.3%). Additionally, 6.7% (14/206) of the *Streptococcus* spp. were resistant to ceftiofur, pirlimycin, and sulfamethoxazole-trimethoprim each. 19 (9.2%) isolates exhibited resistance to more than three antimicrobial classes. No resistance to penicillin G or penicillin-novobiocin was detected for *Streptococcus* spp. All five *Staphylococcus aureus* were resistant to tetracycline, with one isolate resistant to ceftiofur and another to erythromycin. No resistance was detected to penicillin-novobiocin, gentamicin, clindamycin, or cefoxitin (Table 4). Among ten *Escherichia coli* isolates five were wild type based on ECOFFs. However, one *Escherichia coli* showed acquired phenotypic resistance to ciprofloxacin. The single *Klebsiella oxytoca* isolate did not show any acquired phenotypic resistance to any of the agents tested (Table 5).

**Table 4:** Antimicrobial resistance profiles of *Streptococcus* spp. (*n* = 206) and *Staphylococcus aureus* (*n* = 5) isolates from dairy cattle mastitis cases. A dash (−) indicates the antimicrobial was not tested.

Antimicrobial	*Streptococcus* spp *(n = 206)*	*Staphylococcus aureus (n = 5)*
Penicillin G	0	–
Ceftiofur	14 (6.7%)	1 (20%)
Pirlimycin	14 (6.7%)	0
Penicillin-Novobiocin	0	0
Tetracycline	185 (89.8%)	5 (100%)
Gentamicin	–	0
Clindamycin	15 (5.3%)	–
Erythromycin	19 (9.2%)	1 (20%)
Cefoxitin	–	0
Sulfamethoxazole-Trimethoprim	14 (6.7%)	0

**Table 5:** Antimicrobial resistance profiles of *Escherichia coli* and *Klebsiella oxytoca* isolates determined by broth microdilution and interpreted using epidemiological cut-off values (ECOFFs).

Antimicrobial	*Escherichia coli* (*n* = 10)	*Klebsiella oxytoca* (*n* = 1)
Ampicillin	2 (20%)	–
Azithromycin	0	0
Cefotaxime	0	0
Chloramphenicol	1 (10%)	0
Ciprofloxacin	1 (10%)	0
Colistin	0	0
Ceftazidime	0	0
Gentamicin	0	0
Meropenem	0	0
Nalidixic acid	1 (10%)	0
Tetracycline	3 (33.3%)	0
Tigecycline	0	0
Trimethoprim	3 (33.3%)	0

Abbreviations: *n* indicates the number of non-wild type isolates based on broth microdilution results and epidemiological cut-off values (ECOFFs) interpretation.

## 4 Discussion

This study reports limited biosecurity and hygiene practices among smallholder dairy farms, identifies key mastitis risk factors at farm level (floor condition, use of antimicrobials for DCT) and animal level (parity, lactation stage), and characterizes the major bacterial pathogens together with their antimicrobial susceptibility patterns. Our findings demonstrate that udder health management practices on smallholder dairy farms are suboptimal, and are likely shaped by intersecting structural, behavioural, and systemic determinants. Milking equipment, cloth udder towels, and milkers' hands act as high-frequency contact nodes within the herd's infection transmission network. The absence of effective barriers, such as post-milking teat disinfection, practised on fewer than 20% of farms, reduces opportunities for pathogen control and allows infections to persist at endemic levels. Concurrently, inadequate detection of subclinical infections, with only ∼50% of farms using strip cup testing, facilitates silent transmission chains and prolongs pathogen persistence at the herd level. Limited awareness and training gaps on optimal udder health management constrains adoption of evidence-based mastitis control, while structural barriers, such as limited capital investment in biosecurity infrastructure and poor access to extension services, further hinder implementation. At a broader level, several factors contribute to the slow adoption of effective udder health programs. These include a long-standing reliance on traditional milking routines, the emphasis on short-term productivity over preventive hygiene, and plausibly weak incentives within current animal health delivery models to prioritise prevention over treatment. Future interventions should integrate systematic farmer education, strengthened veterinary-farmer engagement, and local and national level benchmarking of udder health indicators i.e. using SCC-based indicators where feasible.

We identified both host and farm level determinants of elevated SCC, serving as proxies for intramammary infection pressure. At the animal level, higher SCC was significantly associated with reduced teat-end clearance (< 30 cm), which likely increases environmental contamination of the teat apex. Physiological factors also played a role: cows in mid- and late-lactation demonstrated elevated SCC compared with early-lactation animals, consistent with cumulative exposure and immunological shifts across the production cycle. Multiparity, particularly in cows with four or more lactations, further increased SCC, underscoring the cumulative risk profile associated with age, parity, and repeated lactation cycles. At the farm level, mastitis risk was strongly associated with environmental and management conditions. Cows from farms with wet floors had significantly higher SCC compared to those from farms with dry floors, highlighting the role of environmental hygiene in pathogen exposure. The observed association between routine use of antimicrobial DCT and elevated SCC may not reflect a causal effect of treatment but rather serve as a marker of underlying disease ecology, with farms adopting DCT likely those already burdened by persistent mastitis infection pressure.

*Streptococcus agalactiae* was the most common pathogen isolated (72.1%). This contrasts with reports from resource-rich dairy production systems, where prevalence is typically below 20%, reflecting global reductions in contagious mastitis pathogens following the implementation of improved milking hygiene protocols (41). However, the rates in our study align with reports from Africa, Asia and South America, where the pathogen accounts for a substantial proportion of mastitis cases, approximately 49%, 40% and 35%, respectively (42). Additional factors may also explain this high proportion i.e. sampling was done between September and December, which are typically characterised by intermittent rainfall and increased humidity, which may promote environmental contamination of the udders and facilitate transmission via milking. Moreover, animals were housed in close proximity and milking was predominately manual, favouring cow-to-cow transmission. Lastly our study used MALDI-TOF MS confirmation, which offers greater sensitivity than earlier studies that relied solely on genus-level culture. *Streptococcus uberis* (10%) was the second most common pathogen. Most of existing studies have not differentiated *Streptococcus* spp. and instead reported only at the genus level. We found that contagious mastitis was the dominant form (73.8%), driven largely by the *Streptococcus agalactiae* prevalence, which reflects deficiencies in optimal udder health practices such as limited adoption of post-milking teat disinfection and the reuse of udder towels that sustain cow-to-cow transmission. Similarly, environmental mastitis associated with *Streptococcus uberis* and other *Streptococcus* spp. accounted for 31.5% of cases, which likely reflects the impact of muddy floors and inadequate biosecurity on the risk of new intramammary infections. Our analysis did not find a significant association between mastitis type and these practices, likely because the overlapping ecology of contagious and environmental pathogens in smallholder systems, where hygiene and housing are uniformly suboptimal, obscures differences between groups.

The mastitis pathogens cultured during this study were generally susceptible to the antimicrobials tested. However, tetracycline resistance was widespread across both *Streptococcus* spp. and *Staphylococcus aureus*, while resistance to all other antimicrobials remained relatively low. The high levels of tetracycline resistance observed are consistent with findings from previous studies in Kenya (43) and other countries (44). In contexts such as ours, where farmers have direct access to veterinary drugs, it is plausible that tetracyclines are preferentially used owing to their low cost and broad-spectrum activity (3). Resistance to penicillin-based antimicrobials and aminoglycosides was absent or low among the isolated *Streptococcus* spp. and *Staphylococcus aureus*. These findings support their effectiveness as first-line treatments for mastitis caused by Gram-positive pathogens. In several countries, such as Scandinavia (45), Estonia (46), and the Netherlands (47), national treatment guidelines recommend the use of simple penicillins as the first-line therapy for common Gram-positive mastitis. However, we do not advocate empirical or blanket use of these antimicrobials. Instead, we emphasise the need for animal health authorities to develop standardised mastitis treatment guidelines informed by local antibiograms, and for animal health professionals to rely on diagnostic testing to guide targeted and responsible antimicrobial therapy.

Existing studies in Kenyan dairy systems have largely relied on CMT for the diagnosis of SCM, with limited incorporation of SCC measurements. The only recent Kenyan study reporting SCC values in smallholder systems documented mean log10 SCC values of 5.3–5.4 cells/mL, corresponding to approximately 2.0–2.5 × 10^5^ cells/mL in rural and peri-urban herds (28). In comparison, the median SCC in our study was lower (1.69 × 10^5^ cells/mL). While the tendency estimates are broadly comparable, a direct comparison is limited as the study was conducted in different counties and production systems compared to our study. These differences may partly explain the variations in the observed SCC distributions.

When comparing CMT with SCC in our study, we observed notable discordance between the two methods; for example, 8% of quarters had SCC values above the diagnostic threshold (≥ 250,000 cells/mL) yet tested negative on the CMT. This discrepancy highlights the inherent limitations of each method. The CMT, while inexpensive and rapid (48), is semi-quantitative and highly dependent on operator judgment and environmental conditions, making it less sensitive to lower SCC elevations. By contrast, SCC is objective and quantitative but not entirely specific, as it can be elevated by non-infectious factors such as parity, lactation stage, or stress (49). These discrepancies have important implications for mastitis diagnosis, surveillance, and management, where CMT may underestimate the true prevalence of SCM. The consequences extend beyond animal health to antimicrobial stewardship. In our settings where CMT is the routine diagnostic tool of choice, misclassification of SCM may contribute to inappropriate antimicrobial use through delayed treatment of undetected infections. A tiered diagnostic approach, with CMT as a primary low-cost field tool supported by confirmatory SCC testing, possibly using emerging portable SCC counters or digital CMT readers, could therefore be considered.

Whilst this study was restricted to farmers affiliated with a single large cooperative, the system is broadly representative of smallholder dairy production in Kenya and much of sub-Saharan Africa, where farmer-owned cooperatives predominate. Financial constraints also precluded SCC testing of all quarters and limited the scope of pathogen characterization, thereby providing only a snapshot of pathogen diversity. In addition, the relatively small number of *Staphylococcus aureus* and Gram-negative isolates reduced the statistical power to draw firm conclusions on their resistance patterns. Potential biases may have arisen from self-reported management practices, which are subject to recall and social desirability bias.

## 5 Conclusion

This study provides integrated quantitative and microbiological evidence on the epidemiology of SCM in smallholder dairy farms in Kenya. Mastitis remains highly prevalent in these systems and is influenced by both host- and farm-level determinants, including parity, lactation stage, teat-end clearance, and suboptimal housing hygiene. The co-occurrence of contagious and environmental pathogens illustrates the dual epidemiology of mastitis in smallholder systems, where persistent reservoirs of infection overlap with continuous environmental exposure, sustaining both a high prevalence and recurrent infection pressure. The generally low resistance observed to penicillin-based antimicrobials supports their prioritisation as first-line therapy for common Gram-positive associated mastitis. However, widespread tetracycline resistance highlights the need for strengthened antimicrobial stewardship. We therefore advocate for the development and implementation of standardised national mastitis treatment guidelines informed by local antimicrobial susceptibility data, alongside greater reliance on diagnostic-supported prescribing. The observed discordance between CMT and SCC further highlights critical gaps in SCM detection, with implications for surveillance accuracy and antimicrobial use decisions. Improving mastitis control in smallholder systems will require a transition from reactive treatment to preventive herd health management, supported by strengthened diagnostics, improved milking hygiene, housing management and sustained veterinary engagement. Embedding these measures within existing milk offtake and cooperative-based support structures offers a feasible pathway to scalable and sustainable adoption.

## Funding sources

This work was supported by the contribution to the Consultative Group for International Agricultural Research Trust Fund (https://www.cgiar.org/funders/) - ’Protecting Human Health Through a One Health Approach’ and the CGIAR Science Program ‘Sustainable Animal and Aquatic Foods (SAAF). Lydiah Kisoo was supported by 10.13039/100010269Wellcome Trust – International Masters' Fellowship award (Ref. 221,483/Z/20/Z). The funders did not participate in any part of the study or publication decision.

## Ethics approval

Licencing for the study was by the National Commission for Science, Technology and Innovation (NACOSTI) (Ref: 566794). Ethical approval to interview farmers was granted by the International Livestock Research Institute (ILRI) Institutional Research Ethics Committee (ILRI-IREC2023–36) and approval to sample cows was granted by ILRI Institutional Animal Care and Use Committee (Ref: ILRI-IACUC2023–13). Written informed consent was obtained from all participating farmers.

## CRediT authorship contribution statement

**Eugine L. Ibayi:** Writing – review & editing, Writing – original draft, Visualization, Validation, Software, Methodology, Investigation, Formal analysis, Data curation, Conceptualization. **Linnet Ochieng:** Writing – review & editing, Writing – original draft, Validation, Methodology, Investigation, Formal analysis, Data curation, Conceptualization. **Lydiah Kisoo:** Writing – review & editing, Validation, Methodology, Investigation, Funding acquisition, Data curation, Conceptualization. **Nanna S. Hansen:** Writing – review & editing, Validation, Investigation. **Rikke L. Jensen:** Writing – review & editing, Validation, Investigation. **Sharon Anyona:** Writing – review & editing, Validation, Investigation. **Abdullahi M. Abdi:** Writing – review & editing, Validation, Investigation. **Arshnee Moodley:** Writing – review & editing, Writing – original draft, Visualization, Supervision, Project administration, Methodology, Funding acquisition, Data curation, Conceptualization. **Dishon M. Muloi:** Writing – review & editing, Writing – original draft, Visualization, Validation, Supervision, Software, Project administration, Methodology, Investigation, Formal analysis, Data curation, Conceptualization.

## Declaration of generative AI and AI-assisted technologies in the writing process

The authors did not use any artificial intelligence-assisted technologies in the writing process.

## Declaration of competing interest

None.

## Data Availability

Data will be made available on request.

## Appendix A Supplementary data

Supplementary material Image 1
