# Supplementary material for: Analysis of somatic cell count, bacteria distribution and antimicrobial susceptibility in lactating dairy cows from small holder dairy farms in Kenya
Source: One Health. 2026 Mar 28;22:101392. doi: 10.1016/j.onehlt.2026.101392 (PMC13091472; doi:10.1016/j.onehlt.2026.101392)
Supplement: Supplementary file 1 — Supplementary material [file mmc1.docx]

**Supplementary material**

**
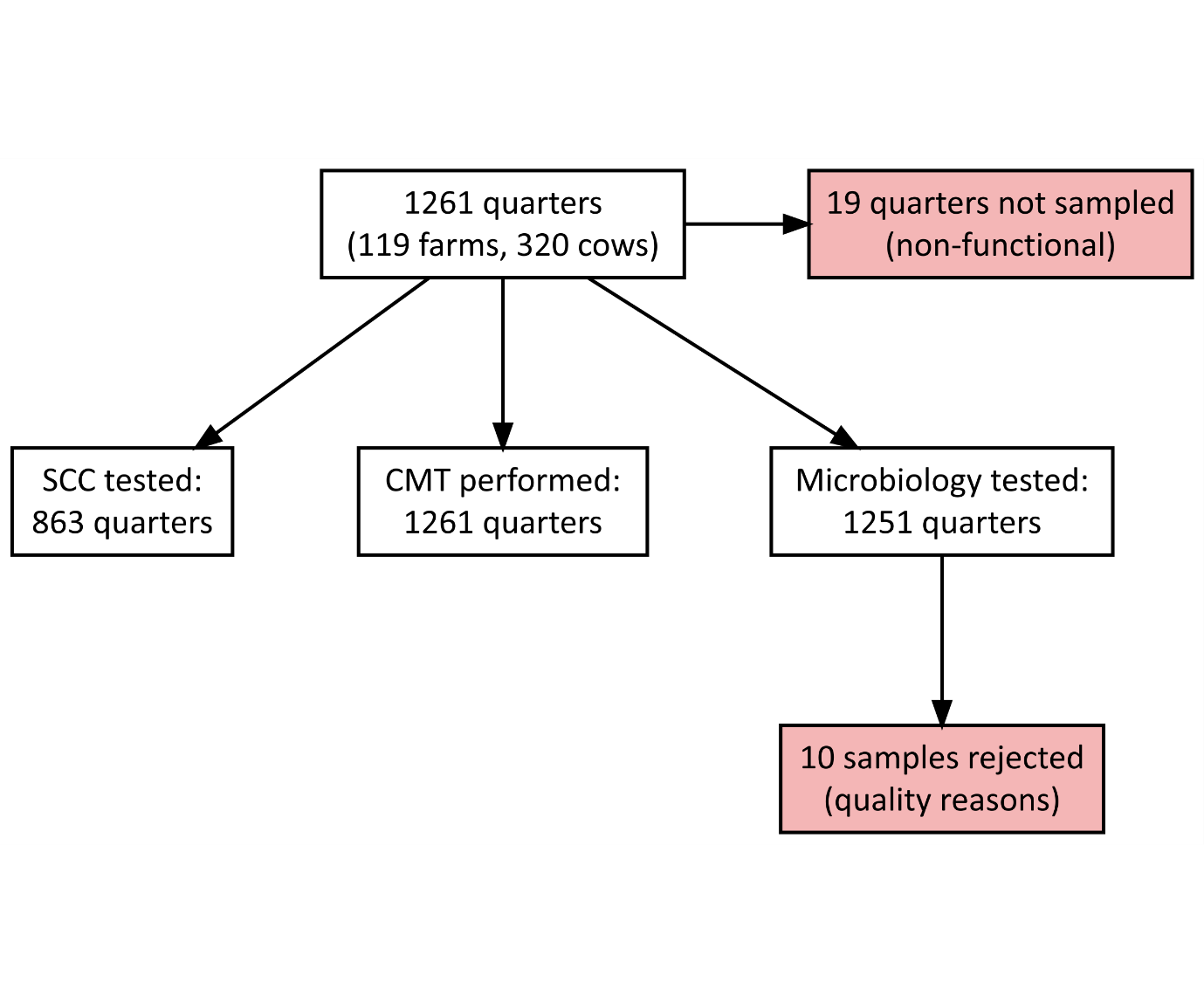
**

Supplementary Figure S1: Flow diagram showing the number of farms, cows, and quarters sampled, and the samples included in the california mastitis test (CMT) and somatic cell count (SCC) analyses.


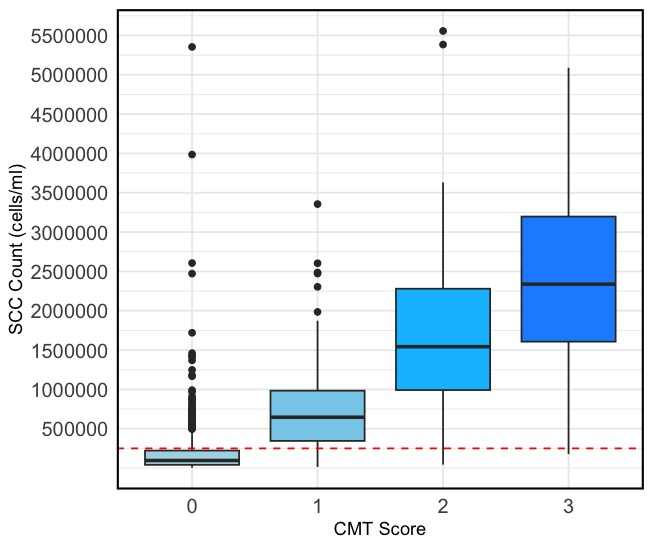


Supplementary Figure S2: Discordance between somatic cell count (SCC) and california mastitis test (CMT) results, with 8% of quarters showing SCC > 250,000 cells/mL but Negative CMT.


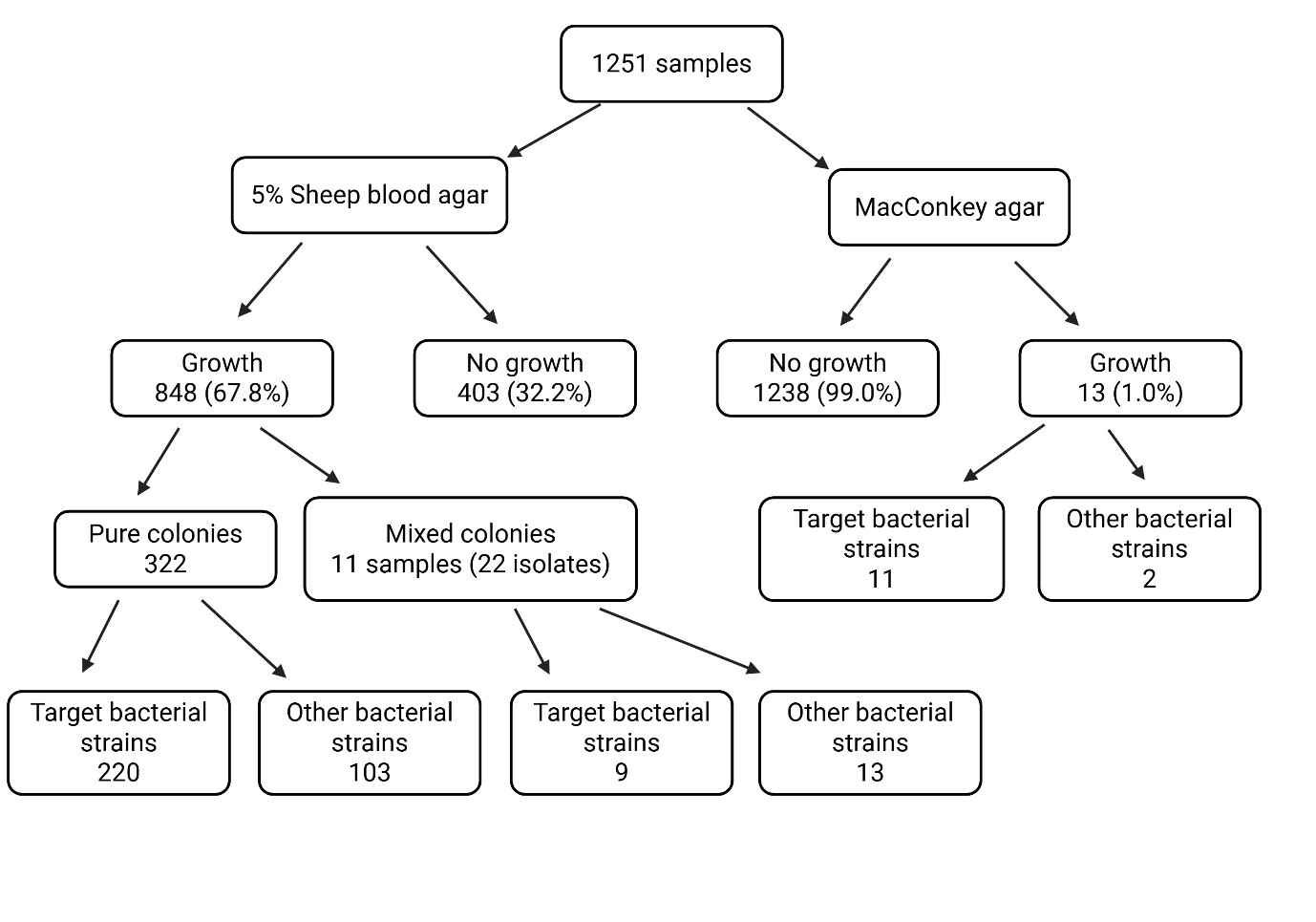


Supplementary Figure S3: Flow diagram showing the isolation process of target bacterial strains from milk samples


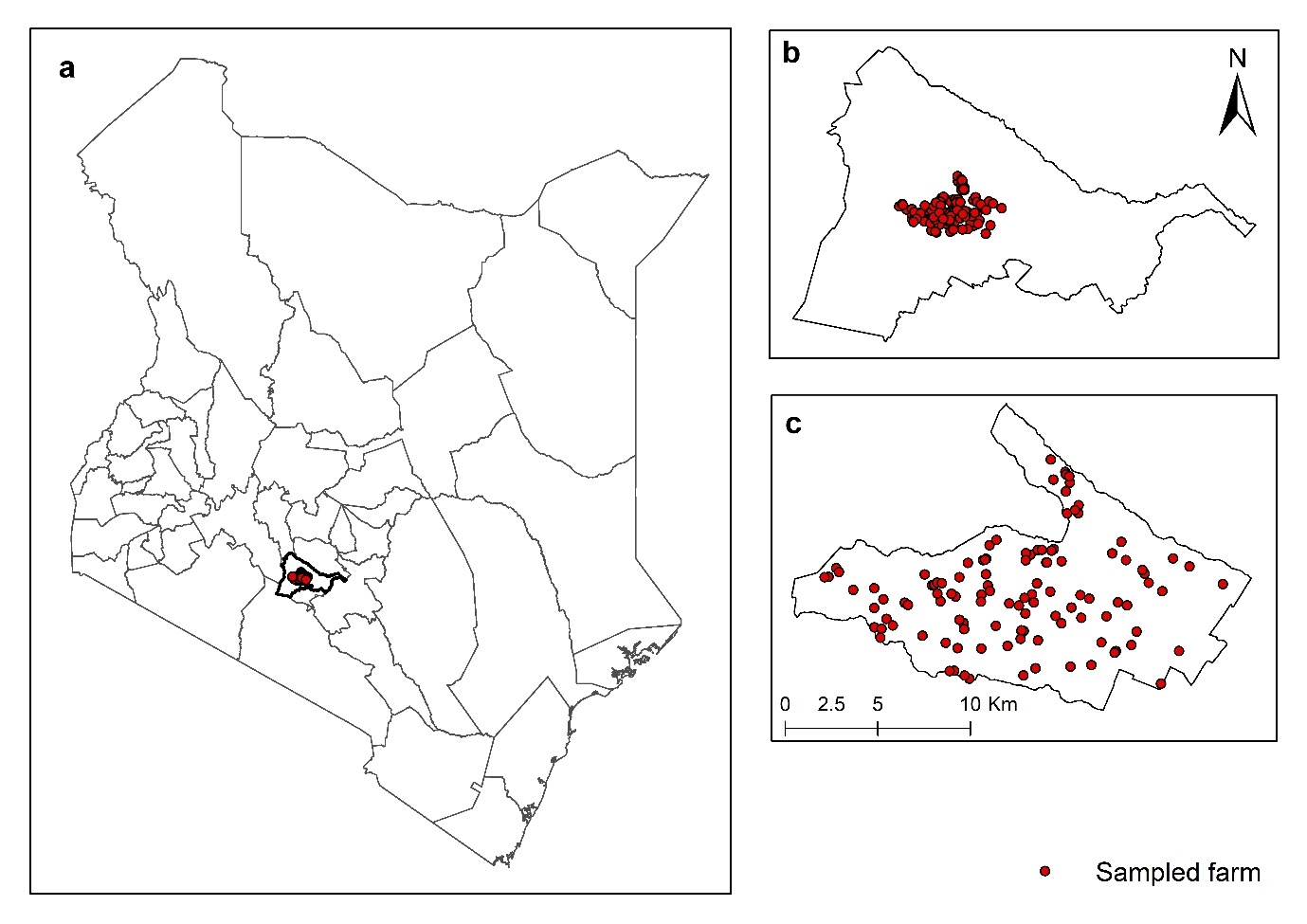


Supplementary Figure S4: Map of the study area showing the sampled farms highlighted in brown. (a) Map of Kenya; (b) Map of Kiambu County; and (c) Map of Githunguri Sub-County.

Supplementary Table S1: Description of explanatory variables and rationale for inclusion

| **Variable group** |  | **Variable** | **Levels, n(%)** | **Rationale (including a reference for the rationale)** |
| --- | --- | --- | --- | --- |
| Farmer characteristics | 1 | Farm owner education level | Secondary (46) 38.7%  tertiary (37) 31.1%  primary (28) 23.5%  no_formal (8) 6.7%  Grand Total (119) | Higher education levels of farm owners are associated with better mastitis management practices |
|  | 2 | Farm owner training (offered by dairy cooperative) | Yes (96) 80.7%  No (23) 19.3%  Total (119) | Training provided by dairy cooperatives enhances farm owners' knowledge and skills, leading to improved mastitis control. |
|  | 3 | Duration of farming (Experience) | Continuous (years) | Longer farming experience is linked to better mastitis management |
|  | 4 | Farm manager education level (offered by dairy cooperative) | secondary (11)39.3%  tertiary (4) 14.3%  primary (12)42.9%  no formal (1)3.6%  Grand Total (28) | Higher education levels of farm managers are linked with ability to easily acquire skills on better mastitis management practices. |
|  | 5 | Farm manager training (offered by dairy cooperative) | Yes (12) 42.9%  No (16) 57.1%  Total (28) | Training farm managers in best practices for mastitis control is crucial for reducing subclinical mastitis. |
|  | 6 | Manager's years in management (Experience) | Continuous (years) | Experienced managers are more effective in implementing mastitis control measures. |
| Herd characteristics | 7 | Herd size | Continuous | Larger herd sizes are associated with higher subclinical mastitis prevalence due to increased challenges in maintaining hygiene and monitoring. |
|  | 8 | Housing type | Semi-open style (113) 95.0%  Closed Style (6) 5.0%  Total (119) | Stall styles are associated with hygiene factors. |
|  | 9 | Number of lactating cows | Continuous | A higher number of lactating cows can complicate mastitis management, leading to increased subclinical mastitis cases. |
|  | 10 | Floor type | Concrete (113) 95.0%  Earthen (6) 5.0%  Total (119) | inadequate flooring can contribute to poor udder hygiene |
|  | 11 | Bedding type | rubber_mats (39) 32.8%  wood shaving/manure/sawdust/soil/sand (80) 67.2%  Grand Total (119) | The type of bedding used can influence the prevalence of subclinical mastitis, with organic bedding materials often harboring more pathogens. |
|  | 12 | Herd cleaning Frequency | Daily (112) 94.1%  Weekly + monthly (7) 5.8%  Total (119) | More frequency of cleaning promoted clean environment that reduces bacterial load |
|  | 13 | Stall wet_dry | Wet (93)78.2%  Dry (26)21.8%  Grand Total 119 | Wet stalls increase the exposure of teat ends to environmental pathogens, raising the risk of subclinical mastitis |
|  | 14 | Separate cubicle | Yes (112)94.1%  No (7)5.9%  Total (119) | Having separate cubicles for cows prevents transmission of pathogens from sick/mastitic to healthy cows, reducing subclinical mastitis risk. |
|  | 15 | Records keeping | Yes (70) 58.8%  No (49) 41.2%  Total (119) | Proper record keeping of mastitis cases, treatments, culling etc. aids in monitoring udder health and implementing control measures, thereby reducing subclinical mastitis. |
| Hygiene practices & biosecurity measures | 16 | Cleanliness score | 2 (172) 53.8%  1 (106) 33.1%  0 (38) 11.9%  Blank (4) 1.2%  Grand Total (320) | Clean cow sheds reduces environmental pathogen load and lowers subclinical mastitis risk |
|  | 17 | Strip-cup to check mastitis | Yes (60) 50.4%  No(59) 49.6%  Total (119) | Stripping foremilk before milking helps detect early signs of mastitis and reduces bacterial load. |
| Milking practices | 18 | Cleaning hands before milking | Yes (115) All | Proper hand hygiene before milking is critical to prevent the introduction of pathogens into the udder. |
|  | 19 | Cleaning udder before milking | Yes (119) - All | Cleaning the udder before milking is essential to remove dirt and bacteria, reducing the risk of subclinical mastitis. |
|  | 20 | Udder cleaning method | Clean with pre-milking dip/water and udder wash/water alone/disinfecting towelette (115) 96.6%  Dry wipe like paper towel (4) 3.4%  Grand Total (119) | Use of udder wash for cleaning the udder is essential to kill bacteria reducing the risk of SCM |
|  | 21 | Drying udder after cleaning method | Dry with disposable/reusable towel (110) 92.4%  Hand dry/do not dry (9) 7.5%  Grand Total 119 | Moisture on the teat ends can allow bacterial entry into the udder |
|  | 22 | Use of pre-milking dip | Yes (24) 20.2%  No (95) 79.8%  Total: 119 | Using a pre-milking teat dip helps reduce bacterial load on teat skin and prevents new intramammary infections, thereby reducing subclinical mastitis risk |
|  | 23 | Use of same towel for all cows for drying | Yes (65) 60.7%  No(42) 39.3%  Total : 107 | Using the same towel for multiple cows can spread pathogens and increase the risk of subclinical mastitis |
|  | 24 | Use of milking lubricant | Yes (115) - All | Using milking lubricants can prevent teat end damage and reduce the risk of mastitis |
|  | 25 | Cows kept upright after milking | Longer than 1 hour (73) 61.3%  30 minutes (38 ) 31.9%  no (8) 6.7%  Grand Total 119 | Keeping cows upright after milking allows the teat sphincter to close, reducing the risk of infection. |
|  | 26 | Use of teat dip or spray after milking | Yes (21) 17.6%  No (98) 82.4%  Grand Total 119 | Using a pre-milking dip helps reduce the bacterial load on the teat skin, preventing new infections |
|  | 27 | Hand washing frequency | btn_each_cow (79) 66.4%  regularly_not_each_cow (34) 28.6%  only when dirty (2) 1.7%  blank 4 (3.4%)  Grand Total 119 | Proper hand hygiene before milking is critical to prevent the introduction of pathogens into the udder. |
|  | 28 | Hand washing method | rinse_water_soap or disinfectant (71) 59.6%  rinse_with_water alone (44) 37.0%  blank (4) 3.4%  Grand Total 119 | Use of a detergent contributes towards better hand hygiene to prevent introduction of pathogens into the udder (S.A.Mekonnen., et al, 2017) <https://doi.org/10.1016/j.prevetmed.2017.06.009> |
|  | 29 | Breed | Ayshire, guernsey or crossbreed 12 (3.8%)  friesian_holstein or Friesian hostein cross 308 (96.2%)  Grand Total 320 | Holstein cows are more susceptible to subclinical mastitis compared to crossbreeds due to genetic factors. |
| Animal characteristics | 30 | Lactation stage | Continuous (months of milk) | Cows in later stages of lactation are at higher risk of subclinical mastitis due to increased somatic cell counts |
|  | 31 | Number of parities | Continuous | Higher parity cows are more prone to subclinical mastitis due to cumulative exposure to pathogens |
|  | 32 | Body condition score | Scale (1-4)  3 209 (65.3%)  2 84 (26.2%)  4 24 (7.5%)  1 3 (0.9%)  Grand Total 320 | Cows with lower body condition scores are more immunocompromised |
|  | 33 | Age | Continous (years) | Older cows are at higher risk of subclinical mastitis due to weakened immune systems and increased exposure to pathogens over time. |
|  | 34 | Teat-floor distance | high_enough(>30cm) 314 (98.1%)  swinging_too_close (<30cm) 6 (1.9%)  Grand Total 320 | Cows with shorter teat-floor distances are more exposed to environmental pathogens, increasing the risk of subclinical mastitis. |
|  | 35 | Milk produced | Continuous (L) | Higher milk production can be associated with increased risk of subclinical mastitis due to stress and metabolic demands |
|  | 36 | History of CM- Mastitis occurrences (within the last 3 months – times of infections) | Continuous | A recent history of clinical mastitis is a strong predictor of subclinical mastitis due to residual infections |
|  | 37 | Mastitis monitoring | no mastitis monitoring (52) 43.7%  irregular monitoring (23) 19.3%  monitor using scc or cmt (44) (37.0%)  Grand Total 119 | Regular monitoring for mastitis, such as somatic cell count testing, helps in early detection and management of subclinical mastitis |
| Animal Health management | 38 | Dry cow therapy | Yes (68) 57.1%  No (51) 42.9%  Grand Total 119 | Proper dry cow therapy, including the use of antibiotics and teat sealants, is crucial for preventing subclinical mastitis in the next lactation |
|  | 39 | Dry cow therapy antibiotic administration | animal health professional 42(61.8%)  farm owner/manager 23 (33.8%)  blank (3) 4.4%  Grand Total 68 | Ensuring that trained personnel administer antibiotics can improve treatment outcomes and reduce subclinical mastitis. |
|  | 40 | Drying off practice | dry cow therapy i.e., every quarter for each cow or some 68 (57.1%)  no dry cow therapy e.g. teat sealant/concentrate withdrawal 51 (42.9%)  Grand Total 119 | Proper dry cow therapy is crucial for preventing subclinical mastitis in the next lactation |
|  |  |  |  |  |

Supplementary Table S2: Minimum inhibitory concentrations (MIC) distributions and EUCAST ECOFF-based classification of Escherichia coli isolates (2025 ECOFFs)

| \| Antibiotics \| MIC Range (mg/L) \| ECOFF (mg/L) \| \| --- \| --- \| --- \| \| Ampicillin \| 4 - > 64 \| 8 \| \| Azithromycin \| 4 - 16 \| 16 \| \| Cefotaxime \| ≤ 0.25 \| 0.25 \| \| Ceftazidime \| ≤ 0.5 \| 1 \| \| Chloramphenicol \| ≤ 8 \| 16 \| \| Ciprofloxacin \| ≤ 0.015 - 0.03 \| 0.06 \| \| Colistin \| ≤ 1 - 2 \| 2 \| \| Gentamicin \| ≤ 0.5 - 2 \| 2 \| \| Meropenem \| ≤ 0.03 \| 0.06 \| \| Nalidixic acid \| ≤ 4 - 8 \| 8 \| \| Sulfamethoxazole \| ≤ 8 - > 1024 \| 64 \| \| Tetracycline \| ≤ 2 - 64 \| 8 \| \| Tigecycline \| ≤ 0.25 - 5 \| 0.5 \| \| Trimethoprim \| ≤ 0.25 - > 32 \| 2 \| |  |  |
| --- | --- | --- | --- | --- | --- | --- | --- | --- | --- | --- | --- | --- | --- | --- | --- | --- | --- | --- | --- | --- | --- | --- | --- | --- | --- | --- | --- | --- | --- | --- | --- | --- | --- | --- | --- | --- | --- | --- | --- | --- | --- | --- | --- | --- | --- | --- | --- |
| Abbreviations: EUCAST = European Committee on Antimicrobial Susceptibility Testing; ECOFFs = Epidemiological Cut-Off values; MIC = Minimum Inhibitory Concentrations |  |  |
|  |  |  |

| Supplementary Table S3: Minimum inhibitory concentrations (MIC) distributions and EUCAST ECOFF-based classification of *Escherichia coli* isolates (2025 ECOFFs)   \| Antibiotics \| MIC Range (mg/L) \| ECOFF (mg/L) \| \| --- \| --- \| --- \| \| Ampicillin \| 32 \| ID \| \| Azithromycin \| 16 \| - \| \| Cefotaxime \| ≤ 0.25 \| 0.25 \| \| Ceftazidime \| ≤ 0.5 \| 0.5 \| \| Chloramphenicol \| ≤ 8 \| ID \| \| Ciprofloxacin \| 0.03 \| 0.06 \| \| Colistin \| ≤ 1 \| 2 \| \| Gentamicin \| ≤ 0.5 \| 2 \| \| Meropenem \| ≤ 0.03 \| 0.125 \| \| Nalidixic acid \| ≤ 4 \| ID \| \| Sulfamethoxazole \| ≤ 8 \| ID \| \| Tetracycline \| ≤ 2 \| 4 \| \| Tigecycline \| ≤ 0.25 \| 1 \| \| Trimethoprim \| ≤ 0.25 \| ID \| |  |  |
| --- | --- | --- | --- | --- | --- | --- | --- | --- | --- | --- | --- | --- | --- | --- | --- | --- | --- | --- | --- | --- | --- | --- | --- | --- | --- | --- | --- | --- | --- | --- | --- | --- | --- | --- | --- | --- | --- | --- | --- | --- | --- | --- | --- | --- | --- | --- | --- |
| Abbreviations: EUCAST = European Committee on Antimicrobial Susceptibility Testing; ECOFFs = Epidemiological Cut-Off values; MIC = Minimum inhibitory concentrations; mg/L = milligrams per liter; ID = Insufficient Data |  |  |
|  |  |  |
|  |  |  |
|  |  |  |

Supplementary Material S1: Mastitis Farm Level Questionnaire

**AMR in Dairy Cattle Baseline Questionnaire**

**1. Owner Survey**

*This form should be administered to the owner of the farm. If the owner is present, or if the owner & manager of the farm are the same person, administer this form first. If the owner is not present, this form should be completed via phone either before or after the manager survey.*

*If the respondent does not know or cannot answer a question, use code “-99” and proceed to the next question.*

**S1. Coversheet**

| 1. Start time | *Autogenerated by the tablet* |
| --- | --- |
| 1. End time | *Autogenerated by the tablet* |
| 1. Today’s date | *Autogenerated by the tablet* |
| 1. Device ID | *Autogenerated by the tablet* |
| 1. GPS | *Captured by tablet* |
| 1. Enumerator ID |  |
| 1. Scan Farm/Household Barcode |  |
| 1. Scan consent form barcode |  |

Who mainly performs the farm work?

*Check any that apply.*

- Farm owner
- Farm worker/manager

**S2. Dairy cattle owner’s section**

**S2.1: Owner's demographics section:**

S2.1q1: Farm owner's gender?

- Male
- Female

S2.1q2: Owner's year of birth?  
*Hint: Between 1930 and 2005* 
s2.1q3: What is the owner's highest education level?

- Primary level
- Secondary level
- Tertiary level
- No formal education

S2.1q4: For how many years have you been an owner of a dairy cattle farm? At this location (and/or at another location farm).  
**S2.2: Owner training section:** 
s2.2q1: have you ever received formal training on dairy cattle production, diseases prevention or disease control in the last 2 years?

- Yes
- No

S2.2q2: What type of training was it? ( if yes selected) 
*hint: (select one)*

- Diploma
- Certificate
- Non-diploma, multiple day course
- One day course
- Other (specify)

S2.2q3: What were you trained on?  (select multiple)

- Increasing production
- Detection of dairy cattle diseases
- Treating dairy cattle diseases
- Biosecurity
- Vaccinations
- Other (specify)

**S3: Employees section:**  
S3q1: Not including yourself, how many people work on or help on the farm?
S3q2: Have the workers ever received formal training on dairy cattle production, disease prevention or disease control in the last 2 years?  
*Hint: May include training by government, NGOs in the last two years (select one)*

- Yes
- Yes, some of them
- None
- Don’t know

S3Q3: What were they trained on? (select multiple)

- Increasing production
- Detection of dairy cattle diseases
- Treating of dairy cattle diseases
- Biosecurity
- Vaccination
- Other (specify)

**S4: Manager Survey (Manager Only) Section:** 
S4: Enumerator reads: now i am going to ask you some questions about yourself and your experience with dairy cattle farming.  
**S4.1: Manager - Demographics:** 
S4.1Q1: Manager's gender?

- Male
- Female

S4.1Q2: Manager's year of birth?
S4.1Q3: What is the manager's highest education level?

- Primary
- Secondary
- Tertiary
- No formal education

S4.1Q4: For how many years have you engaged in dairy cattle management/farming? 
 **S4.2: Manager's Training Section:** 
S4.2Q1: Have you ever received formal training on dairy cattle production, disease prevention or disease control in the last 2 years?

- Yes
- No

 S4.2Q2: What type of training was it? ( select one)

- Diploma
- Non-diploma, multiple day course
- One day course
- Others (specify)

S4.1Q3: How many workers are on the farm?
**S5: Visitors and Farmworkers Section:**  
S5Q1:Is the dairy unit separated from the homestead?

- Yes
- No

S5Q2: Do you have a gated main entrance to the dairy unit?

- Yes
- No

S5Q3: Is there a footbath at the entrance of the dairy unit?

- Yes
- No

S5Q4:Do visitors have access to the dairy unit?

- Always
- Sometimes
- Never

S5Q5: Are they required to wear specific ppe before they enter?

- Always
- Sometimes
- Never

S5Q6: Are visitors obliged to notify you of their presence before entering the dairy unit?  
*Visitor's register*

- Always
- Sometimes
- Never

S5Q7: Is there a separate space available for changing boots and clothes and washing hands/putting on gloves?

- Yes
- No

S5Q8: Are there any farm workers who also work at (or frequently visit) other farms?

- Yes, all.
- Yes, some of them.
- None
- Don't know

**S5Q9: Upon entering the farm, does the farm personnel…**  
Q9.1: Use farm-specific boots?

- Always
- Sometimes
- Never

Q9.1: Use farm-specific boots?

- Always
- Sometimes
- Never

Q9.2: Use farm-specific clothes?

- Always
- Sometimes
- Never

Q9.3: Wash their hands/use gloves before entering**?**

- Always
- Sometimes
- Never

**S6: Farm Characteristics Section:**  
S6Q1: How many cattle have you had on this farm in the past 12 months? All the *All the dairy animals on the farm, including the bulls and the young*
S6Q2: Of the, how many died?
S6Q3.1: So, you have *nan* cattle currently? 
S6Q4: How many of the *nan* cattle are bulls?
S6Q5: How many of the *nan* cattle are lactating?
S6Q6: How many of the *nan* cattle are pregnant cows?
S6Q7: How many of the *nan* cattle are dry cows?  
*Hint: Dry cow: An adult dairy cow that has stopped lactating especially prior to calving (usually 45 - 60 days to calving)*
S6Q8: How many of the nan cattle are heifers?  
*Hint: Heifer: A cow that has not given birth before. Between 3 and 24 months of age* 
S6Q9: How many of the nan cattle are calves? 
*Hint: Calf: A baby cow up to the 3rd month of life.*  
 **S7: Cattle Housing Section:**  
 S7Q1:Do the cows have to regularly pass through a hoof disinfection footbath?

- Always
- Sometimes
- Never
- I do not have

 S7Q2: What do you use in the hoof disinfection bath?

- Copper sulphate
- Zinc sulfate
- Dettol
- Magadi
- Other (specify)

S7Q3: How often do they change them?

- Daily
- Weekly
- Twice a Month
- Monthly
- Other (Specify)

S7Q4: How do they dispose of the <span style =”color:#63ab53”> disinfectant?

- Release into field
- Never change
- Other (Specify)

 S7Q5: Observe: what is the type of housing?

- Semi-open style shed
- Close style shed
- Other (Specify)

S7Q6: Observe: what is the type of floor?

- Concrete
- Earthen

 S7Q7: ASK: How often do you remove the dirt/clean the floor?

- Daily
- Weekly
- Monthly
- > Month

 S7Q8: OBSERVE: Is the floor wet or dry?

- Dry
- Wet

 S7Q9: How many times was the adult stable disinfected in the last 3 months?
S7Q10: OBSERVE/ASK: What type of bedding are you using?

- Wood Shavings
- Sawdust
- Sand
- Rubber
- mats
- None
- Other (Specify)

  S7Q11: ASK: How often do you completely change the beddings?

- Daily
- 2 - 3 times a week
- Once a week
- Monthly
- Other(specify)

  S7Q12: OBSERVE: Each animal housed in a separate cubicle?

- Yes
- No

**S8: Feed and Water Section:**  
 S8Q1: What type of feeds do you use?

- Concentrates
- Hay
- Silage
- Fodder
- Other(specify)

 S8Q2: What is the source of water used on your herd?

- Municipal County water
- Surface water - Dam, Rivers, Lakes
- Rainwater
- Wells and Boreholes

 S9Q1: Does the artificial insemination technician or veterinarian come to the farm?

- Yes
- No

**S9Q2: Upon entering the farm, does the Artificial Inseminator/Veterinarian…** 
Q2.1: Use farm-specific boots?

- Always
- Sometimes
- Never

Q2.2: Use farm-specific clothes?

- Always
- Sometimes
- Never

Q2.3: Wash their hands/use gloves before entering?

- Always
- Sometimes
- Never

**S10: Other Animals' Section:**  
 S10Q1: Is it possible for your cattle to come into contact with animals from other farms?

- Yes
- No

 S10Q2: What other animals are kept on the farm?

- Sheep
- Goats
- Pigs
- Horses
- Poultry
- Dogs
- Cats
- None
- Other
- (Specify)

**S11: Health management Section:**  
S11Q1: Is a register with the animal health data being kept?  
 *Hint: Register: A book or computer programme in which detailed information on health status, past illnesses and treatments of the cattle are stored in an accessible way so that it can be used in decision-making for the sick cattle.*

- Yes
- No

 S11Q2: Within the last 3 months, did you vaccinate your cattle?

- Yes
- No

 S11Q3.1: Why?

- Animals were already vaccinated
- Do not see the value of vaccination
- Could not afford it
- Other (Specify)

S11Q3.2: What disease(s) did you vaccinate against?

- Foot and Mouth Disease (Fotivax)
- East Coast Fever (Muguga Cocktail)
- Contagious Bovine Pleuro-pneumonia (Cantavax)
- Rift Valley fever (Riftovax 19)
- Lumpys Skins Disease (Lumpivax)
- Infectious Bovine Rhinotracheitis
- Bovine Viral Diarrhoea
- Johnes Disease
- Enterotoxaemia (Clostivax)
- Other (Specify)

 S11Q4: In the last 3 months, did your animals ever appear unwell, become sick or die from illness?

- Yes
- No

 S11Q5: How many instances of illnesses did you encounter? 
 S11Q6: What did you do during the sickness?

- Consult Animal Health Service Provider
- Self-treated the animal with antibiotics
- self-treated the animal without antibiotics
- Nothing
- Other

 S11Q7: Was the animal(s) diagnosed/treated by an animal health service provider? 
 *Hint: Veterinary Doctor or Para-veterinarian*

- Yes
- No

  S11Q8: Did the ahsp/yourself submit a sample to the lab for diagnosis?

- Yes
- No

 S11Q9: Why?

- Did not explained why
- Cost of the test is too high
- Long waiting period for a result
- Labs too far away
- Doesn't see benefit of such diagnostic services
- Doesn't have confidence in test results
- Other (Specify)

**S12: Calving management Section:**  
  S12Q1: When does the separation of the calf from the dam take place?

- Within an hour of the calf's birth
- No separation, the calf remains with the dam as a suckling calf
- As soon as the born calf is seen.
- Other (Specify)

 S12Q2: Do give colostrum to your calves?

- Always
- Sometimes
- Neve**r**

**S13: Udder Health and Milking Hygiene Section:**  
 S13Q1: Are the cows milked with a milking machine or manually?

- Machine
- Manually

S13Q2: What is the average number of milkings per cow per day on your farm?
 s13q3: Do you clean udder/teats before milking?

- Yes
- No

 S13Q4: How do you clean the udder/teats before attaching the milking units/hand milking?

- Dry wipe like paper towel and serviettes
- Clean with pre-milking teat dip
- Clean with water and udder wash
- Clean with water (without udder wash)
- Clean with commercially available wet disinfecting towelette (e.g., ReadyWipe)
- I do not clean the teats
- Other (Specify)

S13Q5: How do you dry the udder/teats of different cows?

- Disposable paper towels
- Reusable cloth towel
- I do not dry teats/udder
- Other (Specify)

S13Q6: When do you clean the teat cup?

- After each animal
- After each milking session
- No cleaning of teat cup

 S13Q7: Do you use pre-milking dip?

- Yes
- No

 S13Q8: Do you use rubber or silicone teat cup liners?

- Rubber teat cup liner
- Silicone teat cup liner
- None

S13Q9: After how many months are the teat cup liners replaced?  
S13Q10: Do you clean/disinfect the teat cup liner before a milking session?

- Yes
- No

S13Q11: Do you clean/disinfect the teat cup liner after a milking session?

- Yes
- No

 S13Q12: Do you use the same towel to dry udder/teats of different cows?

- Yes
- No

S13Q13: Do you strip milk from each quarter into a strip cup to check for mastitis?  
*Hint: What do you do before starting milking?*

- Yes
- No

 S13Q14: In which situation(s) do you typically fore-strip milk?

- On every cow, at every milking
- On cows that are suspected of mastitis
- On cows that have clinical mastitis
- On cows with elevated Somatic Cell Counts (SCC)
- Other (Specify)

S13Q15: During milking do you ensure that you have removed almost all the milk from the teats?

- Yes
- No

S13Q16: Are the teats disinfected after the teat cups are removed?

- Yes, with a dip.
- Yes, with a spray
- No

S13Q17: Are cows kept upright for a period after milking?

- Yes, for 30 minutes to an hour
- Yes, for longer than an hour
- No

S13Q18: Do you use a teat dip or spray after milking?

- Yes
- No

S13Q19: Are the cows milked in a specific order?  
*Hint: SCC: Somatic Cell Count*

- Cows with mastitis are milked last.
- Cows with mastitis are milked first
- Other (Specify)

S13Q20:Do you wash hands before milking?

- Yes
- No

 S13Q21: How often do you wash your hands?

- Between each cow
- Regularly, but less often than between each cow
- Only if they are dirty

 S13Q22: How do you wash your hands?

- Rinse with water
- Rinse in a disinfecting solution
- Rinse with water and soap
- Other (Specify)

 S13Q23: Do the milkers wear gloves?

- Always
- Sometimes
- Never

S13Q24: Do you apply/use milking jelly/lubricant/salve on your hands before milking?

- Yes
- No

 S13Q25: How often do the people milking cows in your herd get trained/retrained to use the same milking routine?

- Only when they are hired/start milking
- Less than once a year
- Once a year
- More than once a year
- Not trained?

**S14: Dry Cow Management Section:**  
 S14Q1:Do you administer antibiotic at the end of the production cycle (end of milking period)?  
 *Hint: Dry cow therapy: intramammary infusion with an antibiotic(s) at the end of the production cycle. Dry period is the period prior to calving, usually 45 - 60 days, and milking normally has ceased.*

- Yes
- No

 S14Q2: What antibiotic(s) was administered?

- Tylosin
- Sulfamethoxazole
- Doxycycline
- Oxytetracycline
- Tetracycline
- Colistin
- Amikacin
- Amoxicillin
- Amoxicillin + Clavulanic acid
- Ampicillin Ampicillin + Sulbactam
- Apramycin
- Aspoxicilin
- Avilamycin
- Bacitracin
- Baquiloprim
- Benethamine penicillin
- Benzylpenicillin
- Benzylpenicillin procaine
- Bicozamycin
- Carbadox
- Carbomycin
- Cefacetrile
- Cefalexin
- Cefalonium
- Cefalotin
- Cefapyrin
- Cefazolin
- Cefoperazone
- Cefquinome
- Ceftiofur
- Ceftriaxone
- Cefuroxime
- Chlortetracycline
- Ciprofloxacin
- Cloxacillin
- Danofloxacin
- Dicloxacillin
- Difloxacin
- Dihydrostreptomycin
- Enramycin
- Enrofloxacin
- Erythromycin
- Florphenicol
- Flumequin
- Fortimycin
- Fosfomycin
- Framycetin
- Fusidic acid
- Gamithromycin
- Gentamicin
- Gramicidin
- Hetacillin
- Josamycin
- Kanamycin
- Kitasamycin
- Lasalocid
- Lincomycin
- Maduramycin
- Marbofloxacin
- Mecillinam
- Miloxacin
- Mirosamycin
- Monensin
- Nafcillin
- Nalidixic acid
- Narasin
- Neomycin
- Nitarsone
- Norfloxacin
- Nosiheptide
- Novobiocin
- Ofloxacin
- Olaquindox
- Oleandomycin
- Orbifloxacin
- Ormetoprim
- Ormetoprim + sulfadimethoxine
- Oxacillin
- Oxolinic acid
- Paromomycin
- Penethamate (hydroiodide)
- Phenethicillin
- Phenoxymethylpenicillin
- Phthalylsulfathiazole
- Pirlimycin
- Polymixin
- Rifampicin
- Rifaximin
- Roxarsone
- Salinomycin
- Sarafloxacin
- Sedecamycin
- Semduramicin
- Spectinomycin
- Spiramycin
- Streptomycin
- Sulfachlorpyridazine
- Sulfadiazine
- Sulfadimethoxine
- Sulfadimidine (sulfamethazine, sulfadimerazine)
- Sulfadoxine
- Sulfafurazole
- Sulfaguanidine
- Sulfamerazine
- Sulfamethoxine
- Sulfamethoxypyridazine
- Sulfamonomethoxine
- Sulfanilamide Sulfapyridine
- Terdecamycin Thiamphenicol
- Tiamulin
- Ticarcillin
- Tildipirosin
- Tilmicosin
- Tobicillin
- Tobramycin
- Trimethoprim
- Trimethoprim + sulphonamide
- Tulathromycin
- Tylvalosin
- Valnemulin
- Virginiamycin
- None
- Do not know
- Other (Specify)

 S14Q3: Who administered the antibiotic(s)? Government registered Veterinarian Farm owner Farm worker

- Agrovet worker
- Other AHSPs (Specify)

 S14Q4:Do you disinfect the teat end before intramammary antibiotic(s) infusion?

- Always
- Sometimes
- Never

S14Q5: Do you use teat sealant?

- Yes
- No

**S15: Mastitis at the Farm Level Section:**  
S15Q1: Approximately how many cattle have had mastitis within the last 3 months?  
S15Q2: Within the last 3-month period have you culled any cow(s) because of mastitis?

- Yes
- No

 S15Q3: How many cows were culled?
 S15Q4: Did any cow(s) die from mastitis in the last 3 month period?

- Yes
- No

S15Q5: How many cows died? 
S15Q6: Which of the following procedures do you regularly use to monitor mastitis in your herd?

- I review the individual somatic cell counts of my cows
- I use the California mastitis test to detect sub-clinical mastitis
- I take milk samples, then culture and/or test for PCR to detect subclinical mastitis
- I do not have a regular program to monitor subclinical mastitis
- None

 S15Q7: How do you identify cows with mastitis?

- I visually tag them (leg tags, hip spraying etc.)
- I keep a record of them
- Other (Specify)

S15Q8: When drying off cows in your herd, which best describes your usual practice?

- Dry cow intramammary antibiotic in every quarter of every cow
- Dry cow intramammary antibiotic used for some, but not all cows/quarters
- Dry cow intramammary antibiotic is never used
- Internal teat sealant in every quarter of every cow
- Internal teat sealant is used for some, but not all cows/quarters
- Internal teat sealant is never used
- Withdrawal of concentrate or highly nutritious feeds

 S15Q9: What method do you use to diagnose and/or monitor and test for mastitis on your farm?

- California Mastitis Test
- Somatic Cell Count
- Bacterial Culture
- Visual Examination of the milk
- Clinical Signs
- Other (Specify)

 S15Q10: What do you do when a cow(s) has mastitis?

- Call an Animal Health Service Provider
- Self-treat with Antibiotic
- Self-treat without Antibiotic
- Cull (Sell, Slaughter, Euthanize)
- None
- Other (specify)

 S15Q11: Select antibiotics:

- Tylosin
- Sulfamethoxazole
- Doxycycline
- Oxytetracycline
- Tetracycline
- Colistin
- Amikacin
- Amoxicillin
- Amoxicillin + Clavulanic acid
- Ampicillin Ampicillin + Sulbactam
- Apramycin
- Aspoxicilin
- Avilamycin
- Bacitracin
- Baquiloprim
- Benethamine penicillin
- Benzylpenicillin
- Benzylpenicillin procaine
- Bicozamycin
- Carbadox
- Carbomycin
- Cefacetrile
- Cefalexin
- Cefalonium
- Cefalotin
- Cefapyrin
- Cefazolin
- Cefoperazone
- Cefquinome
- Ceftiofur
- Ceftriaxone
- Cefuroxime
- Chlortetracycline
- Ciprofloxacin
- Cloxacillin
- Danofloxacin
- Dicloxacillin
- Difloxacin
- Dihydrostreptomycin
- Enramycin
- Enrofloxacin
- Erythromycin
- Florphenicol
- Flumequin
- Fortimycin
- Fosfomycin
- Framycetin
- Fusidic acid
- Gamithromycin
- Gentamicin
- Gramicidin
- Hetacillin
- Josamycin
- Kanamycin
- Kitasamycin
- Lasalocid
- Lincomycin
- Maduramycin
- Marbofloxacin
- Mecillinam
- Miloxacin
- Mirosamycin
- Monensin
- Nafcillin
- Nalidixic acid
- Narasin
- Neomycin
- Nitarsone
- Norfloxacin
- Nosiheptide
- Novobiocin
- Ofloxacin
- Olaquindox
- Oleandomycin
- Orbifloxacin
- Ormetoprim
- Ormetoprim + sulfadimethoxine
- Oxacillin
- Oxolinic acid
- Paromomycin
- Penethamate (hydroiodide)
- Phenethicillin
- Phenoxymethylpenicillin
- Phthalylsulfathiazole
- Pirlimycin
- Polymixin
- Rifampicin
- Rifaximin
- Roxarsone
- Salinomycin
- Sarafloxacin
- Sedecamycin
- Semduramicin
- Spectinomycin
- Spiramycin
- Streptomycin
- Sulfachlorpyridazine
- Sulfadiazine
- Sulfadimethoxine
- Sulfadimidine (sulfamethazine, sulfadimerazine)
- Sulfadoxine
- Sulfafurazole
- Sulfaguanidine
- Sulfamerazine
- Sulfamethoxine
- Sulfamethoxypyridazine
- Sulfamonomethoxine
- Sulfanilamide Sulfapyridine
- Terdecamycin Thiamphenicol
- Tiamulin
- Ticarcillin
- Tildipirosin
- Tilmicosin
- Tobicillin
- Tobramycin
- Trimethoprim
- Trimethoprim + sulphonamide
- Tulathromycin
- Tylvalosin
- Valnemulin
- Virginiamycin
- None
- Do not know
- Other (Specify)

 S15Q12: What do you do to prevent and control mastitis on your farm?

- Dry Cow Therapy
- Ensure Clean Environment
- Teat dipping/spraying with disinfectant
- Segregate/eliminate infected animals
- Screen new animals for mastitis
- Milking hygiene and technique Selecting cow(s) with good udder conformation
- Other (Specify)

 S16Q1: Who provides you with veterinary extension services? 
 S16Q2: Please what is his/her contact number 
 S17:Do you have any comment about the household

Supplementary Material S2: Mastitis Farm Level Questionnaire

S1: Coversheet Section.

| S1Q1: Enumerator ID: |  |
| --- | --- |
| S1Q2: Scan Farm/Household Barcode |  |

S2.1: Sampled Animals' Section:

S2.1Q1: How many animals have been sampled on the farm. (*please note that 3 animals are to be sampled per farm*)

S2.2: Sampled Animal Section:

S2.2Q1: What is the breed of the sampled cow?

- Friesian/Holstein-Friesian
- Ayrshire
- Jersey
- Guernsey
- Fleckvieh
- Crossbreed
- Other (Specify)

S2.2Q2: What is the age of the animal? [in years. Ask for estimate if the age is unknown].
S2.2Q3: How many litres of milk does she averagely produce daily?
S2.2Q4: The number of times the animal has calved /given birth?

S2.2Q5: What is the body condition score of the animal? BCS 1: vertebrae prominent in the loin and rump, individual bones easily visible. BCS2: Easily feel individual bones, vertebrae in loin and rump area less visually distinct. BCS 3: Vertebrae in the loin and rump area appear rounded, individual bones not distinct. BCS 4: Loin and rump areas appear flat. BCS 5: Loin and rump not visible and difficult to feel individual vertebrae.

- 1
- 2
- 3
- 4
- 5

S2.2Q6: Observe: select the animal cleanliness score:

- 0
- 1
- 2

S2.2Q7: On how many occasions has the animal suffered/been diagnosed with mastitis in the past 3 months?

 S2.2Q8: What did you do when the animal had mastitis?

- Consult Animal health service provider.
- Self-treated the animal with antibiotics
- Nothing
- Other (specify)
  S2.2Q9: Was an antibiotic(s) administered during the sickness?
- Yes
- No

S2.3: Antibiotic therapy section:

 S2.3Q1: Which antibiotic(s) was administered?

            [List of antibiotics]

  S2.3Q2: Why did you decide to use this antibiotic?

- Previous knowledge
- Advice from AHSPs
- Advice from Agrovet
- Advice from pharmaceutical representatives
- Advertisement
- Non AHSPs advice(friend/family)
- Other (Specify)

S2.3Q3: Where did you buy the antibiotic?

- Agrovet
- Private animal health services provider
- Government animal health services provider
- Friends or neighbours
- Pharmaceutical sales representatives
- Other (Specify)

S2.3Q4: Who administered the antibiotic?

- Government registered veterinarian
- Farm owner
- Farm worker
- Agrovet worker
- Other AHSPs

S2.3Q5: On how many occasions was the antibiotic administered? S2.3Q6: Please take the front photo of the antibiotic

S2.3Q7: Please take the back photo of the antibiotic

S2.4Q1: OBSERVE: How close are the teat ends to the floor distance of the animals?

- Swinging too close(30cm)
- High enough (>30cm)

S2.4Q2: How long have you been milking this animal? in months

S2.4Q3:OBSERVE: Are there any mastitis symptoms on the udder?

- Warm udder
- Reddening
- Watery milk
- Clot/flakes in milk
- Swelling of the udder
- Pain on palpation
- Hardness in teats/udder
- Pus in milk
- None
- Other

S2.5: California Mastitis Test (CMT) Results Session:

S2.5Q1: Left front quarter cmt results

- Negative (0)
- Weakly Positive (1)
- Positive (2)
- Strongly Positive (3)
- No sample

S2.5Q2: Left rear quarter cmt results

- Negative (0)
- Weakly Positive (1)
- Positive (2)
- Strongly Positive (3)
- No sample

S2.5Q3: Right front quarter cmt results

- Negative (0)
- Weakly Positive (1)
- Positive (2)
- Strongly positive (3)
- No sample

S2.5Q4: Right rear quarter cmt results

- Negative (0)
- Weakly Positive (1)
- Positive (2)
- Strongly positive (3)
- No sample

S2.6: Samples Barcodes Section:

S2.6Q1: ILRI milk sample - left front quarter

S2.6Q2: Githunguri milk sample - left front quarter

S2.6Q3: Type the code for Githunguri milk sample - left front quarter

S2.6Q4: ILRI milk sample - left rear quarter

S2.6Q5: Githunguri milk sample - left rear quarter

2.6Q6: Type the code for Githunguri milk sample - left rear quarter

S2.6Q8: Githunguri milk sample - right front quarter

S2.6Q9: Type the code for Githunguri milk sample - right front quarter

S2.6Q10: ILRI milk sample - right rear quarter

S2.6Q11: Githunguri milk sample - right rear quarter

S2.6Q12: Type the code for Githunguri milk sample - right rear quarter

S3: What is the method(s) of disposing milk from an animal on antibiotic therapy?

- Household Consumption
- Sell
- Feeding the calves
- Feeding the dogs (and cats -animals in the homestead)
- Discard elsewhere (environment, toilet etc)
- Other (Specify)

S4: Do you have any comments about the household?
